# Prediction Model for Deformation of Concrete Dam Based on Interpretable Component Decomposition and Integration

**DOI:** 10.3390/s26082495

**Published:** 2026-04-17

**Authors:** Feng Han, Chongshi Gu

**Affiliations:** 1 College of Water Conservancy and Hydropower Engineering, Hohai University, Nanjing 210098, China; hfsgghz@126.com; 2 State Key Laboratory of Hydrology-Water Resources and Hydraulic Engineering, Hohai University, Nanjing 210098, China; 3 College of Hydraulic Science and Engineering, Yangzhou University, Yangzhou 225009, China

**Keywords:** concrete dam, interpretable prediction model of deformation, CEEMDAN, BiLSTM network, SHAP method

## Abstract

**Highlights:**

**What are the main findings?**

- An integrated decomposition-optimization prediction framework (CEEMDAN-BKA BiLSTM) was proposed, which significantly improved the accuracy and stability of concrete dam deformation prediction by separating interpretable deformation components and modeling them separately.
- The introduction of SHAP method quantifies the contribution of each input factor, achieving interpretability analysis of the prediction results of the “black box” model.

**What are the implication of the main findings?**

- Overcoming the limitations of conventional models in comprehensively describing complex components, it provides a more reliable and robust analysis technique for dam safety monitoring.
- It solves the problem of difficult identification of key factors in traditional models and provides a new diagnostic method for intelligent operation and maintenance of dams that combines high accuracy and transparency.

**Abstract:**

A dam deformation prediction method based on interpretable component decomposition and integration is proposed to address the problems of weak interpretability, difficult identification of key factors, and insufficient accuracy in the prediction model of deformation monitoring values of concrete dams due to multiple factors such as environmental loads and time factors. This method first strips the temporal component from the original sequence to obtain the castration sequence. Furthermore, complementary ensemble empirical mode decomposition with adaptive noise (CEEMDAN) is used to decompose and reconstruct it into environmental load components and residual terms. In the process of deformation prediction, based on the characteristics of each deformation component, logarithmic functions, bidirectional long short-term memory (BiLSTM) networks optimized by The Black-Winged Kite Algorithm (BKA), and cloud models are used to fit and predict the temporal components, environmental load components, and residual terms, and the final prediction results are obtained through integration. At the same time, the SHAP (SHapley Additive exPlanations) method is introduced to quantify the contribution of input factors to enhance the interpretability of the model. Case study shows that the model outperforms the comparison model in both prediction accuracy and trend tracking ability, effectively improving the reliability of prediction results and significantly increasing the interpretability of deformation prediction, providing a more reliable analysis technique for dam deformation safety monitoring.

## 1. Introduction

As critical hydraulic infrastructures, dams necessitate periodic safety assessments to safeguard downstream communities. Continuous monitoring and evaluation of dam displacement and deformation are indispensable for elucidating operational states and long-term behavioral trends. Such practices constitute essential measures for ensuring structural safety and stability throughout the dam’s service life, while simultaneously serving as vital instruments for validating original design assumptions, appraising construction quality, and comprehending the evolution of key performance parameters [1,2]. Propelled by advances in modern engineering, dam safety monitoring has evolved into an interdisciplinary framework that integrates sensor technologies, advanced data analytics, and structural mechanics. Consequently, the scope of surveillance has expanded from conventional surface displacement measurements to coupled multi-physics analyses encompassing stress distributions, seepage regimes, and thermal fields within the dam body. The safety monitoring system of concrete dams endows operators with an exceptionally robust real-time surveillance capability that is indispensable for safeguarding structural integrity. This capability enables the expeditious detection of anomalous phenomena within the dam body, thereby facilitating rapid assessment of current operational safety and supporting timely intervention. Moreover, the system provides a critical lens through which the evolving service conditions of hydraulic structures—under the combined action of adverse loading scenarios and multifarious influencing factors—can be rigorously investigated and characterized [3,4,5,6]. The displacement field of a concrete dam exhibits a pronounced non-linear dependence on reservoir-level fluctuations, and thermally induced annual cyclic deformation can account for 30–50% of the total annual deformation. Nevertheless, displacement series are modulated by environmental forcings and, therefore, manifest pronounced spatiotemporal complexity, posing significant challenges to accurate forecasting. These series comprise both deterministic components—driven by hydrostatic pressure, thermal loads, and aging effects—and stochastic constituents arising from material creep and foundation deterioration. Consequently, enhancing the predictive accuracy of dam displacement remains a central concern in the scholarly literature.

Dam deformation is widely recognized as one of the most critical factors affecting operational safety [2]. By establishing quantitative relationships between deformation monitoring data and their governing influences, the safety state of concrete dams can be rigorously assessed [7,8]. Conventional approaches—encompassing statistical, deterministic, and hybrid modeling paradigms—have been extensively employed for deformation surveillance. Among these, statistical regression techniques underpin the earliest global dam deformation monitoring models, which attribute observed deformation to hydrostatic, thermal, and temporal effects [9,10,11]. Driven by the inherent limitations of conventional predictive frameworks, a growing body of literature has turned to machine-learning paradigms for dam displacement forecasting. Early attempts incorporated shallow architectures such as BP neural networks [12], artificial neural networks [13], extreme learning machines [14], and least-squares support-vector machines [14]. In parallel, deep-learning techniques have gained prominence. Li et al. [15] enhanced the Harris Hawks optimizer and fused it with a quasi-recurrent neural network to achieve superior predictive accuracy. Zhang et al. [16] optimized the hyperparameters of extreme gradient boosting via the sparrow search algorithm, yielding an efficient displacement model. Lu et al. [17] employed empirical mode decomposition for signal preprocessing and integrated the gray wolf optimizer with a long short-term memory network to construct an advanced settlement-prediction model. Zhang et al. [18] proposed an adaptive-optimized relevance vector machine coupled with random forests for concrete dam deformation forecasting. Su et al. [19] combined support-vector machines with chaos theory and particle swarm optimization to optimize both input selection and parameter calibration, establishing a robust safety prediction framework. In comparison with traditional empirical formulations, intelligent monitoring paradigms—ranging from neural networks and extreme learning machines to multi-algorithmic fusion architectures—demonstrate marked improvements in predictive accuracy, thereby enabling more reliable assessments of dam safety. Additionally, the decomposition of raw displacement series effectively attenuates sequence complexity, which in turn enhances forecasting performance. Li [20] employed empirical mode decomposition (EMD) to preprocess displacement data, subsequently optimized a genetic-algorithm-based model, and aggregated the component-wise predictions; Li et al. [21] integrated ensemble empirical mode decomposition (EEMD) with a support-vector regressor for deformation analysis. Notwithstanding these advances, classical EMD suffers from mode-mixing artifacts that compromise the fidelity of localized feature extraction. Although EEMD mitigates mode-mixing by iterating the decomposition with added white noise, the procedure incurs a substantial computational burden. To overcome these limitations, enhanced variants such as complementary ensemble empirical mode decomposition with adaptive noise (CEEMDAN) have been proposed in recent years [22]. However, the application of CEEMDAN in the decomposition of concrete dam deformation time series is relatively limited, and further research is needed on the adaptability and reliability of this method in its deformation decomposition field, to improve the level of concrete deformation sequence decomposition by leveraging the advantages of this method. Beyond deformation prediction, advanced monitoring techniques have also been applied to understand fracture evolution in quasi-brittle materials under extreme thermal conditions, such as granite subjected to cyclic thermal shocks and liquid nitrogen cooling. Although these studies focus on rock mechanics, the signal processing and machine-learning methodologies employed therein provide valuable insights for dam deformation analysis [23,24]. Beyond dam deformation prediction, machine-learning and hybrid deep-learning techniques have also been successfully applied to other areas of structural engineering. For instance, recent studies have explored the automated design and optimization of concrete beams reinforced with stainless steel [25], developed serviceability-based deflection models for RC beams with stainless steel reinforcement [26], and employed inverse machine learning for the design of perforated beams [27]. Although these studies focus on different structural elements and design problems, the data-driven methodologies and optimization frameworks employed therein provide valuable insights that can be adapted to dam deformation modeling. Inspired by these advances, the present study adopts a hybrid decomposition–optimization–prediction framework to improve the accuracy and interpretability of concrete dam deformation forecasting.

However, deep-learning models rely on multiple nonlinear transformations to achieve end-to-end prediction, which renders them inherently opaque: the semantic link between the latent feature space and the input variables is severed, the evidential basis of the output remains untraceable, anomaly detection results are difficult to interpret, and the contribution of individual monitoring indicators to anomalous behavior cannot be quantitatively assessed [28]. To address these interpretability deficits, Lundberg et al. [29] introduced in 2017 the SHAP (SHapley Additive exPlanations) framework. Rooted in cooperative game theory, this post hoc explanation technique disentangles model decision logic by attributing precise influence scores to each monitoring indicator during anomaly detection, thereby substantially enhancing model transparency and diagnostic reliability. This method is introduced in this article to quantitatively analyze the various input factors of environmental load components, time-dependent components, and residual components of concrete dam deformation, sort the influence and importance of each factor on deformation, and improve the interpretability of each input factor in the deformation prediction model.

Accordingly, the environmental load component, time-dependent component, and residual component of concrete dam deformation are decomposed, fitted and predicted separately, and then merged and integrated. First, use CEEMDAN decomposition to obtain the environmental load components of deformation, reduce data complexity, and then use BKA to optimize the BiLSTM model to establish a prediction model for the environmental load components of dam deformation. To address the random nature of high-frequency residual terms, a cloud model is used to fit and predict the residual components, using logarithmic functions to fit and predict the time-dependent components of dam deformation, and integrating the predicted values of the three components to obtain the deformation prediction results of the dam. Finally, through case analysis, the interpretable component decomposition ensemble prediction model proposed in this paper has higher prediction accuracy compared to other models. The research on the interpretability of SHAP-driven anomaly detection results not only clarifies the dynamic causes of dam displacement anomalies, but also facilitates a more comprehensive analysis of dam safety status and promotes the cognitive upgrading of intelligent operation and maintenance systems.

## 2. Interpretable Component Decomposition Method for Concrete Dam Deformation

### 2.1. Deformation Component of Concrete Dam

Usually, the total deformation of a dam is decomposed into three main interpretable components: water pressure component, temperature component, and time-dependent component [30]. The component characterization relationship of concrete dam deformation is:

(1) $$\delta =\delta_{H}+\delta_{T}+\delta_{\theta }$$

where δ is the deformation of the dam. $\delta_{H}$, $\delta_{T}$ and $\delta_{\theta }$ are respectively the water pressure component, temperature component and aging component.

The water pressure component is the deformation of the dam body and foundation caused by the rise and fall of the reservoir water level. For concrete dams, the deformation caused by reservoir water pressure is generally linearly related to the upstream water depth H, which can be approximately fitted as an exponential polynomial of the upstream water depth *H*. The mathematical expression for the water pressure component is:

(2) $$\delta_{H}=\sum_{i=1}^{4}a_{i}H^{i}$$

where *H* is the upstream water depth corresponding to the monitoring day; and *ai* is the regression coefficient of the characteristic factor of the water pressure component.

The temperature component is the thermal expansion and contraction deformation caused by the periodic changes in the temperature field of the dam concrete and foundation rock mass, as well as long-term temperature differences. The mathematical expression for temperature component is:

(3) $$\delta_{T}=b_{0}+\sum_{i=1}^{m}\left(b_{i}sin\frac{2\pi it}{365}+b_{i+1}\cos\frac{2\pi it}{365}+b_{i+2}\sin\frac{2\pi it}{365}\cos\frac{2\pi it}{365}\right)$$

where *t* is the cumulative number of days from the monitoring date to the starting date; *b*_i_, *b*_i+1_ and *b*_i+2_ are the regression coefficients of temperature component characteristic factors; *m* is generally taken as 1 or 2, where *m* = 1 represents a one-year cycle and *m* = 2 represents a six-month cycle. In this article, *m* is taken as 1.

The time-dependent component is an irreversible deformation that accumulates slowly over time, reflecting long-term effects such as material rheology, geological consolidation and compression, and structural damage accumulation. The mathematical expression for the time effective component is:

(4) $$\delta_{\theta }=c_{0}+c_{1}\ln\theta$$

where *θ* is the cumulative number of days *t* from the monitoring date to the starting date divided by 100. *C*_0_ is a constant term. *C*_1_ is the regression coefficient of the temporal component characteristic factor.

In this study, the term “aging” refers to the time-dependent component of dam deformation, which physically includes the creep and shrinkage of concrete, the creep of foundation rock mass, and the accumulation of structural damage. These factors collectively lead to an irreversible increase in deformation over time. The logarithmic function (Equation (4)) is adopted to fit the aging component because it captures the typical behavior of rapid initial change followed by gradual stabilization. Regarding the relationship between elastic modulus increase and deformation: during concrete hardening, the elastic modulus increases, which reduces elastic deformation under the same load; however, creep and shrinkage continue to produce additional time-dependent deformation. The aging component model in this paper comprehensively reflects these combined effects, rather than separately quantifying the change in elastic modulus.

In addition, for dam deformation monitoring data, in addition to the deformation components mentioned above, monitoring errors are also included. Usually, monitoring errors exist in the form of high-frequency noise. Because in addition to peeling off the aforementioned components, it is also necessary to separate and obtain high-frequency measurement errors $\varepsilon_{t}$. According to the expressions of the above components, the deformation monitoring values of the dam can be characterized by a multivariate statistical model as follows:

(5) $$\begin{array}{ll} \delta_{t} & =a_{0}+\sum_{i=1}^{4}a_{i}H^{i}+\sum_{i=1}^{m}\left(b_{i}\sin\frac{2\pi it}{365}+b_{i+1}\cos\frac{2\pi it}{365}+b_{i+2}\sin\frac{2\pi it}{365}\cos\frac{2\pi it}{365}\right)\\ & +c_{1}\ln\theta +\varepsilon_{t} \end{array}$$

where $\delta_{t}$ represents the deformation monitoring value of the dam. *a*_0_ is a constant term, which combines *b*_0_ and *c*_0_. The meanings of other symbols are consistent with the above expression symbols.

Due to the distinct properties of the three components of dam deformation, directly incorporating them into the model for prediction will lead to a decrease in prediction accuracy. According to Equation (5), the regression coefficients of each component can be obtained, which can then be used to predict the changes in the temporal components. Considering that c0 is a constant term, the deformation monitoring value minus the variation in the time-dependent component can be obtained as follows:

(6) $$\delta_{t}-c_{1}\ln\theta =a_{0}+\delta_{H}+\delta_{T}+\varepsilon_{t}$$

The meaning of the symbols in Equation (6) is consistent with the meaning of Equations (1)–(5) in the above expression.

In this study, multiple regression analysis was conducted on the deformation monitoring data of B12-IP-01 point (Equation (5)), and regression coefficients for time-dependent components were obtained. The estimated coefficient C1 is 5.86, which is statistically significant at the *p* < 0.01 level. A positive C1 value indicates that the deformation over time increases with time, while the logarithmic form ensures a gradually decreasing growth rate, which is consistent with the typical creep and consolidation behavior of concrete dams. Compared with the range of time-varying coefficients reported in similar engineering studies (approximately 0.5–7.0), the obtained C1 = 5.86 falls within a reasonable range, indicating that the extracted time-varying components have physical significance and meet the expected requirements for dam deformation analysis.

### 2.2. CEEMDAN Deformation Decomposition Method

CEEMDAN was developed to overcome the mode-mixing limitation inherent in EMD and to suppress the non-negligible reconstruction error that arises in EEMD when the ensemble size is small [31]. The key innovation lies in injecting a controlled, finite-amplitude adaptive noise directly into the EMD/EEMD framework. Specifically, the algorithm begins by adding Gaussian white noise *n*(*i*) of amplitude *ε*_0_ to the original signal *x*, producing an ensemble of perturbed signals

*x*(*i*) = *x* + *ε*_0_ × *n*(*i*), *i* = 1, 2, …, *N* (7)

where *i* indexes the noise realization.

Each *x*(*i*) is then subjected to standard EMD, yielding its first intrinsic mode function *IMF*1 (*i*). The ensemble average of the N realizations of *IMF*1 (*i*) gives the first CEEMDAN mode *IMF*1. Finally, the first residual is computed as *r*_1_:

(8) $$IMF1=\frac{1}{N}\sum_{i=1}^{N}IMF1_{i}$$

(9) $$r_{1}\left(i\right)=x(i)-IMF1$$

The procedure is then reiterated on the updated residual. Gaussian white noise of amplitude is added to *r*_1_(*i*) to generate *r*_2_, and the ensemble EMD yields *IMF*2.

Until the residual components obtained can no longer be decomposed, at this point, all the components decomposed by CEEMDAN and the final trend term are obtained. Then the original sequence is divided into *K* subsequences and a residual sequence.

(10) $$x=\sum_{k=1}^{K}IMFk+R(i)$$

where *IMFk* represents the *k* modal component; *R*(*i*) represents the final residual.

From this, it can be concluded that the residual *R*(*i*) is the measurement error of the deformation monitoring value, and the other components are the environmental load components of the deformation.

## 3. A Dam Deformation Prediction Model Based on Solvability Component Integration

### 3.1. Black-Winged Kite Algorithm (BKA)

The Black-Winged Kite Algorithm (BKA), introduced by Wang et al. [32] in 2024, is a novel population-based meta-heuristic inspired by the migratory and predatory behaviors of the black-winged kite (Elanus caeruleus). By abstracting the bird’s capacity for hovering, sudden diving, and adaptive relocation, BKA not only emulates the species’ remarkable hunting and navigation strategies, but also encodes its high responsiveness to environmental fluctuations and target positions. This biomimetic formulation endows the algorithm with robust dynamic-search capabilities, enabling effective adaptation to varying optimization landscapes. The algorithm proceeds through three sequential stages.

(1) Population initialization stage

The initial population is randomly generated. The individual position Xi of each black-winged kite represents a potential solution in the solution space:

(11) $$X_{i}=X_{\min}+rand(0,1)(X_{\max}-X_{\min})$$

where *rand* is a random value between 0 and 1, and X_min_ and X_max_ are the boundaries of the search space.

(2) Attacking (local exploitation)

As a predator of small grassland mammals and insects, the black-winged kite continuously analyses real-time wind speed during flight to modulate its wing- and tail-attack angles, thereby stabilizing its hovering posture for unobtrusive prey surveillance before executing a rapid stoop. This composite strategy encompasses multiple attack behaviors that serve global exploration and search objectives. The corresponding mathematical model is specified as follows:

(12) $$X(t+1)=\left\{\begin{array}{l} X(t)+n(1+\sin(r))\times X(t)\;p<r\\ X(t)+n\times (2r-1)\times X(t)\;\mathrm{else}\\ n=0.05\times exp{\left(-2\times t/T\right)}^{2} \end{array}\right.$$

where *p* is a constant; *T* represents the total number of iterations; and *t* represents the number of completed iterations.

(3) Migration stage

Avian migration is a complex behavior shaped by environmental variables such as climate variability and resource availability. In response to seasonal shifts, numerous species undertake south-bound movements from northern latitudes during winter to secure more favorable survival conditions and abundant resources. These movements are typically coordinated by leaders whose navigational proficiency is critical to the flock’s success. Within the algorithmic abstraction, if the fitness of the current population falls below that of a randomly selected population, the incumbent leader is deemed unsuitable and relinquishes its role, rejoining the migratory swarm. Conversely, should the current population exhibit superior fitness, the leader continues to guide the flock toward the destination. The Black-Winged Kite Algorithm (BKA) incorporates this leadership mechanism by adaptively selecting competent leaders, thereby ensuring efficient convergence toward resource-rich regions. The associated position-update equation is expressed as

(13) $$X(t+1)=\left\{\begin{array}{l} X(t)+C\left(0,1\right)\times \left(X(t)-L_{t}^{j}\right)\;F_{i}<F_{ri}\\ X(t)+C\left(0,1\right)\times \left(L_{t}^{j}-m\times X(t)\right)\;\mathrm{else}\\ m=2\times sin\left(r+\pi /2\right) \end{array}\right.$$

where *F_i_* represents the fitness value at the current position; *F_ri_* stands for Random Position Fitness Value; *C*(0, 1) is a random number generated in the Cauchy distribution.

### 3.2. BiLSTM Algorithm

**Figure 1:**
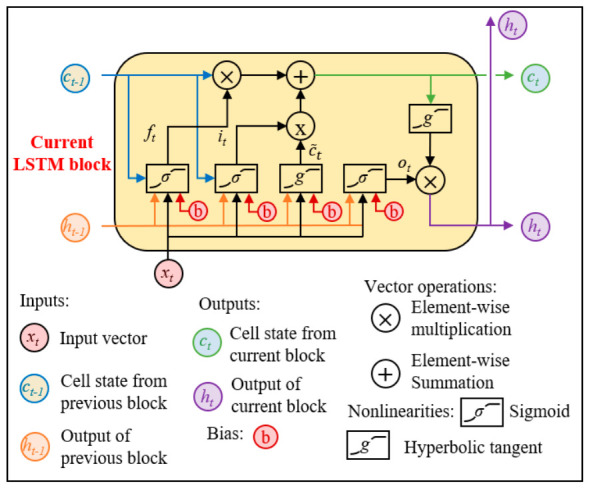
Structure diagram of LSTM.

Unlike standard recurrent neural networks (RNNs), the long short-term memory (LSTM) architecture endows the model with a dynamic capacity—via its distinctive gating units—to selectively retain or discard information along the temporal dimension. This design enables the effective capture of intricate temporal patterns and the management of long-range dependencies, thereby alleviating the vanishing-gradient problem that commonly arises when traditional RNNs process lengthy sequences. The structure diagram of LSTM is shown as Figure 1.

As shown in Figure 1, the gate structure can be used to control the flow and storage of information, thereby achieving the learning of long-term dependencies in sequence data [33,34,35]. Equations (14)–(19) briefly describe the update of the LSTM layer.

(14) $$i_{t}=\sigma \left(V_{i}X_{t}+W_{i}h_{t-1}+b_{i}\right)$$

(15) $$f_{t}=\sigma \left(V_{f}x_{t}+W_{f}h_{t-1}+b_{f}\right)$$

(16) $${\tilde{c}}_{t}=\tanh\left(V_{c}x_{t}+W_{c}h_{t-1}+b_{c}\right)$$

(17) $$c_{t}=f_{t}⊗c_{t-1}+i_{t}⊗{\tilde{c}}_{t}$$

(18) $$o_{t}=\sigma \left(V_{0}x_{t}+W_{0}h_{t-1}+b_{0}\right)$$

(19) $$h_{t}=o_{t}⊗\tanh(c_{t})$$

where *x_t_* is the input data at time *t*, $V_{*}$ and $W_{*}$ are weight matrices, $h_{*}$ is the number of hidden units, $b_{*}$ is the linear bias of the fully connected layer, *σ* and *tan h* are the activation functions of sigmoid and tanh, respectively; *i_t_*, *f_t_*, *c_t_* and *o_t_* are input gates, forget gates, storage units, and output gates, respectively. ⨂ represents element-wise multiplication between vectors. Finally, the output *h_t_* is calculated based on the information stored in the output gate and storage unit.

The BiLSTM extends this framework by integrating two independent LSTM layers that operate in opposite temporal directions. Whereas a vanilla LSTM relies exclusively on past information for sequential prediction, BiLSTM concurrently leverages contextual information from both the forward and backward propagation paths by fusing their respective memory cells. This bidirectional fusion markedly enhances data-utilization efficiency and model expressiveness, ultimately yielding improved predictive accuracy.

**Figure 2:**
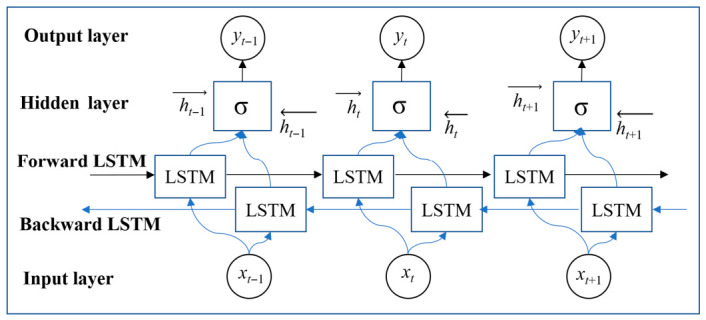
Structure diagram of BiLSTM.

For long-sequence forecasting tasks such as displacement, which are characterized by high-resolution sampling, conventional predictive models frequently exhibit sub-optimal performance. The BiLSTM-based approach proceeds by first feeding the displacement series into a forward LSTM layer whose hidden states are caught. In parallel, a backward LSTM layer processes the temporally reversed copy of the same series; its hidden representations are subsequently concatenated—or otherwise fused—with the forward outputs to yield the final prediction. This bidirectional learning mechanism in Figure 2 shows how BiLSTM works, it enables a more comprehensive exploitation of temporal dependencies, resulting in superior predictive accuracy and enhanced performance in time-series analysis.

The hyperparameters of BiLSTM network need to be determined, and the time cost of manual parameter tuning is relatively high, while it cannot guarantee the optimal learning efficiency. Therefore, BKA is used to optimize the hyperparameters of the BiLSTM network, and a BKA-BiLSTM deformation prediction model is established to solve the problems of manual parameter tuning. The input factors of the BKA-BiLSTM model include water pressure factors *H*, *H*^2^, *H*^3^, *H*^4^, temperature factors sin 2πit/365, cos 2πit/365, sin (2πit/365) cos (2πit/365), and the output variable of the BKA-BiLSTM model is the environmental load component of deformation.

### 3.3. SHAP Method

Due to the multiple influencing factors of each component of dam deformation, in order to increase the interpretability of the prediction model, many only complete end-to-end data prediction. This paper introduces the SHAP method to analyze the influencing factors of each deformation component.

SHAP is a model interpretation method based on the Shapley value of game theory, whose core principle is to regard the predicted results of the model as the benefits of a ‘cooperative game’, and each feature is the ‘player’ participating in the game [36]. It calculates the average marginal contribution of a feature to the prediction results among all possible feature combinations (alliances) with or without a certain feature, in order to fairly distribute the “benefits” and obtain the SHAP value of each feature. This method has key properties such as local accuracy (the sum of SHAP values of features plus the baseline value is exactly equal to the model prediction value) and consistency. It cannot only explain how the prediction of a single sample is pushed up or down by different features, but also reveal the global behavior of the model by summarizing the SHAP values of all samples, such as feature importance ranking and its positive or negative correlation with the prediction results.

In this article, the predicted deformation value of the dam is the benefit of the ‘cooperative game’, and the influencing factors of each deformation component are the ‘players’ participating in the game. With the help of SHAP method, the importance ranking of local and global features of various influencing factors of water pressure component, temperature component, and aging component can be carried out, achieving the analysis of the contribution of each factor to dam deformation, further revealing the controlling factors of dam deformation, and providing technical guidance for dam deformation safety monitoring.

### 3.4. Implementation Framework and Process of Dam Deformation Prediction Model

The specific implementation steps of the concrete dam deformation prediction model based on integrated interpretable components are as follows:

Step 1: Preprocess the dam displacement data and environmental characteristic values, use statistical models to extract the time-dependent components of dam deformation, and use logarithmic function models for prediction.

Step 2: Subtract the time-dependent component from the dam deformation monitoring values to obtain the trend component of the deformation. Use CEEMDAN to decompose its components into subsequences of different frequency bands [IMF1, IMF2, ..., IMF*K*]. The IMF*K* in the high-frequency band represents the error of deformation monitoring values, while the IMF in other frequency bands represents the environmental load component of deformation.

Step 3: Divide the processed component data into training set, testing set, and prediction set in chronological order for subsequent operations.

Step 4: Build a BiLSTM learning network, input environmental load component data as the training set, and use the BKA to optimize the hyperparameters of the model. Set MAE as the fitness function to construct a prediction model for environmental load components in BKA-BiLSTM.

Step 5: Use cloud models to fit and predict the residual term IMF*_K_* in the high-frequency range and obtain the error distribution function of the monitoring values.

Step 6: Input the prediction set data into the constructed BKA-BiLSTM prediction model, predict the environmental component of dam displacement, and combine the prediction results of the temporal component and residual term to obtain the prediction results of the CEEMDAN-BKA-BiLSTM model, and analyze the prediction results.

Step 7: Using the SHAP method, rank the importance of the influencing factors of dam deformation and conduct explanatory analysis on each deformation component.

To ensure reproducibility, the key hyperparameters and optimization settings are detailed as follows. The BiLSTM network consists of two hidden layers with 64 and 32 neurons, respectively, and a dropout rate of 0.2 after each layer. The learning rate, number of neurons in each layer, dropout rate, and batch size are optimized by the BKA. The search ranges are: learning rate ∈ [0.0001, 0.01], neurons in the first layer ∈ [32, 128], neurons in the second layer ∈ [16, 64], dropout rate ∈ [0.1, 0.5], and batch size ∈ [16, 64]. The BKA is run with 50 iterations and a population size of 30. The fitness function is the validation MAE obtained from 5-fold cross-validation, and the optimization stops early if no improvement in the best fitness is observed for 10 consecutive iterations. After optimization, the final selected hyperparameters are: learning rate = 0.001, neurons (first layer) = 64, neurons (second layer) = 26, dropout = 0.2, and batch size = 31. Due to limitations in computing resources and time, this study did not introduce regularization terms or cross-validation strategies for multi-objective fitness functions, and instead used MAE as the fitness function.

**Figure 3:**
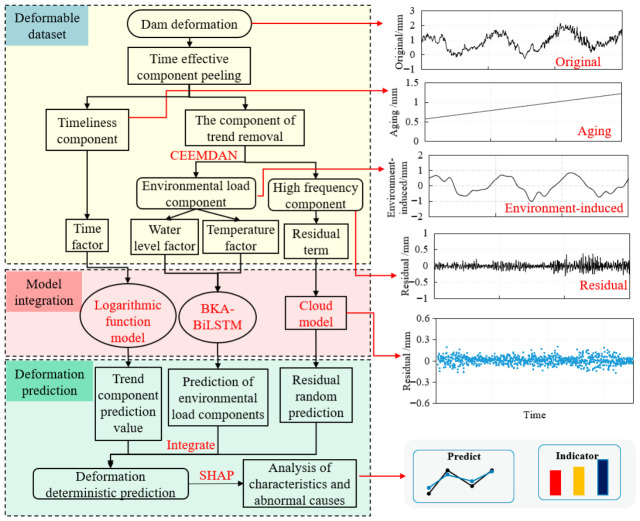
Flowchart of dam deformation prediction model.

In summary, the implementation process of the deformation prediction model for concrete dams is shown in Figure 3.

## 4. Case Study

### 4.1. Project Overview

**Figure 4:**
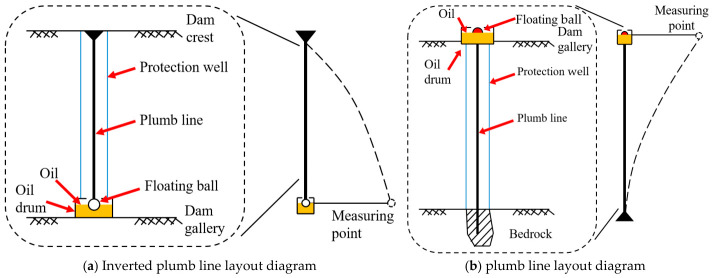
Working principle diagram of plumb line.

A certain hydropower station is mainly used for power generation, with a project grade of Class II large (2) type. The main building level is Class 2, and the hub retaining dam is a concrete dam. One set of inverted plumb system is installed on each side of the dam shoulder as the working base point for the tensioning line, namely B1-IP-01 on the left bank and B15-IP-01 on the right bank. At the same time, a set of inverted plumb lines and plumb lines, namely B12-PL-01 and B12-IP-01, are respectively installed in the dam section of the factory building and the observation room of the foundation gallery. The working principle diagram of the plumb lines is shown in Figure 4. Figure 4a is the layout diagram of the inverted plumb line, and Figure 4b is the layout diagram of the plumb line. The instrument type is a vertical coordinate meter. The data are collected automatically once per day, with a measurement accuracy of ±0.1 mm.

**Figure 5:**
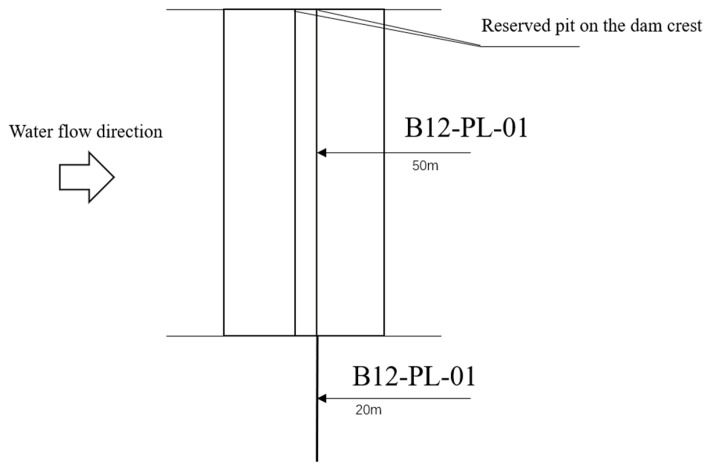
Plumb lines layout of dam Section 12.

Select the monitoring data of inverted plumb line and plumb line at measuring point B12-IP-01 in the 12th dam section as the research object to construct a prediction model. The plumb lines layout is shown in Figure 5.

**Figure 6:**
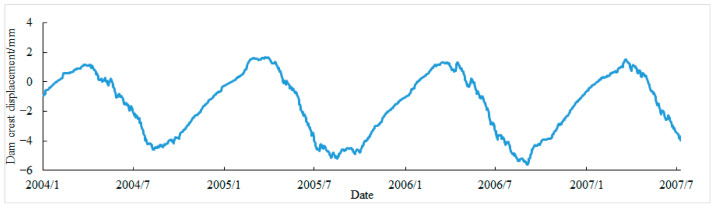
The process line of the monitoring data for dam crest displacement.

After using interpolation method to supplement missing values, 1226 data points were obtained. To facilitate the construction of the training model and prediction, the process line of the monitoring data is shown in Figure 6. Divide the dataset in chronological order, taking 982 sets of data as the training set, 122 sets of data as the testing set, and 122 sets of data as the prediction set.

### 4.2. Time-Sensitive Component Extraction and Detrended Deformation Decomposition

**Figure 7:**
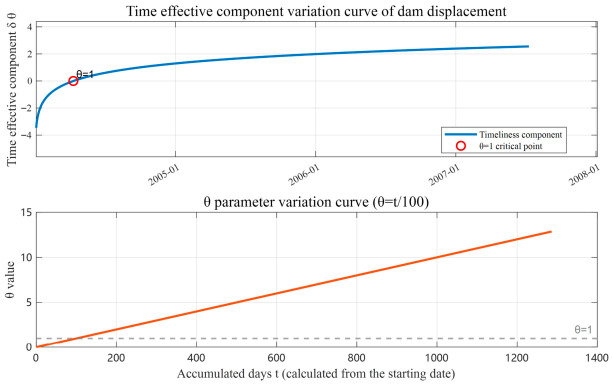
Time effective component curve.

Using Equation (5) to perform multiple regression analysis on the deformation monitoring values of the dam, and fitting the time-dependent components in logarithmic function form, the fitted curve is shown in Figure 7.

**Figure 8:**
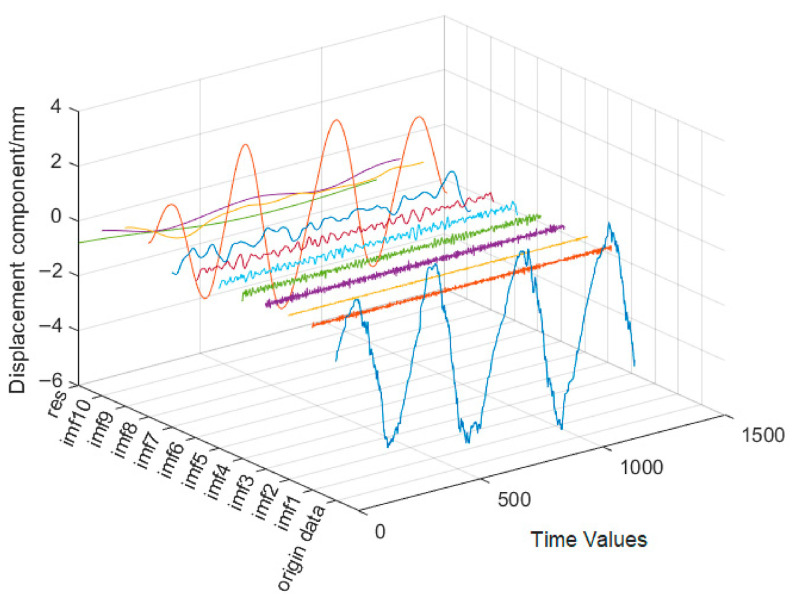
IMF subsequences after decomposition of deformation sequence.

The deformation trend component sequence is obtained by subtracting the time-dependent component from the measured deformation values of the dam. The CEEMDED method is used to decompose its modal, resulting in 10 subsequences and a residual sequence, as shown in Figure 8.

### 4.3. Deformation Prediction Results and Analysis

**Figure 9:**
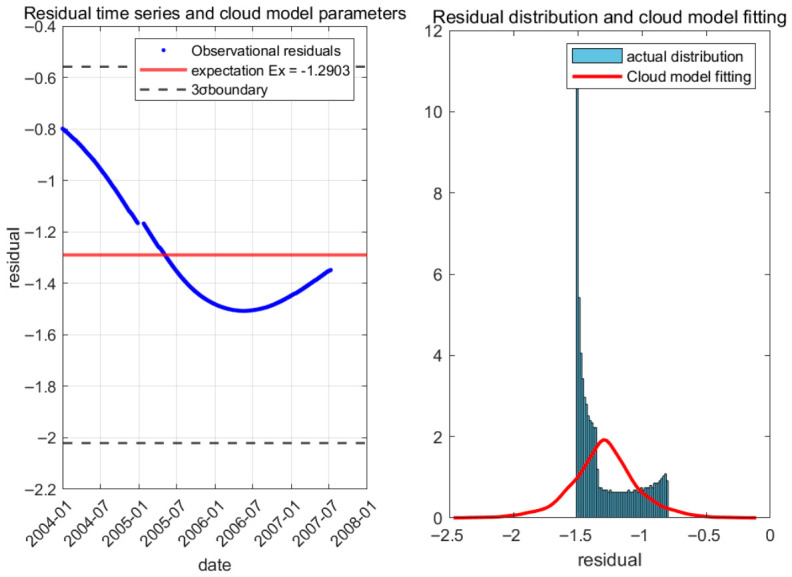
Prediction value of deformation residual.

High-frequency residual terms have significant randomness and ambiguity, and often do not satisfy the assumption of stationarity. The ARIMA model requires the sequence to be stationary or transformed into stationary through differencing, while the Gaussian process requires a pre-set covariance function and requires a large amount of computation for large-scale data. In contrast, cloud models can describe the overall distribution of residuals through the three numerical features of expectation, entropy, and super entropy, without strict distribution assumptions, and are more suitable for the common “limited sample, irregular distribution” residual sequences in engineering monitoring. This advantage has been widely recognized in the fields of water conservancy engineering and structural health monitoring. Therefore, this article uses cloud models to calculate residuals. The cloud model was used to fit and predict the residual random term of deformation, and the results are shown in Figure 9.

Then, the BKA BiLSTM model is used to predict the environmental load, and the final prediction data is obtained by combining the temporal components and residual terms. To verify the predictive performance of the CEEMDED-BKA BiLSTM model, CNN, LSTM, BKA BiLSTM models, and POA BiLSTM models based on Pelican optimization algorithm were constructed and compared for prediction. The processed B12-IP-01 measurement point training set data and corresponding feature values were input into each model for model training. The fitting accuracy of the training set is shown in Table 1.

**Figure 10:**
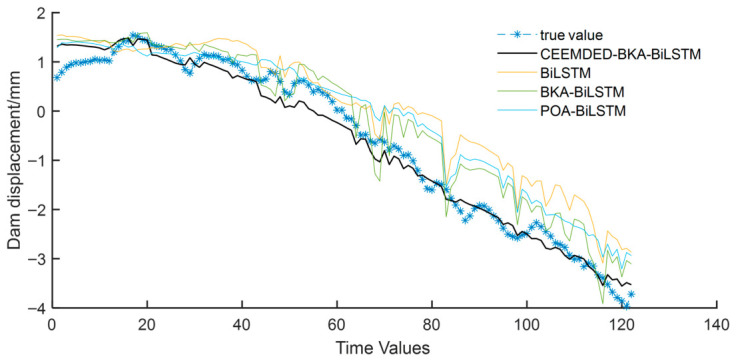
Prediction results of various models at B12-IP-01 measurement point.

From Table 1, it can be seen that the fitting effect of each model is good, indicating that the constructed model is reasonable and can be used for subsequent prediction. Among them, CEEMDED-BKA BiLSTM has the best fitting effect. Input the deformation measurement point prediction set data into the trained model for prediction, and use multiple evaluation indicators to quantify the model’s prediction performance. The evaluation indicators of the model prediction set are shown in Table 2, and the comparison between the prediction curves of each model and the true values is shown in Figure 10.

From Figure 10, it can be seen that the CEEMDED-BKA-BiLSTM model is closest to the measured values of dam deformation in terms of both magnitude and variation trend compared to other models, and has the best prediction performance, indicating that this model can significantly improve the predictive ability of dam deformation. Furthermore, according to the evaluation indicators of prediction accuracy in Table 2, it can be seen that the MAE, MAPE, MSE, and RMSE indicators of the CEEMDED-BKA-BiLSTM model are the smallest compared to other models. Specifically, compared to the evaluation metrics of several other models, the MAE of the CEEMDED-BKA-BiLSTM model decreased by 50.95–66.36%; MAPE decreased by 7.16–37.72%; MSE decreased by 80.89–88.95%; RMSE decreased by 50.54–66.75%; R2 increased by 8.62–26.09%. This indicates that the predicted values of the model proposed in this article are closest to the true values, with minimal numerical fluctuations and the most stable prediction performance.

In addition to the evaluation metrics of the model, due to the fast iteration efficiency advantage of the BKA optimization algorithm, the CEEMDED-BKA-BiLSTM model also has a significant improvement in running time compared to other optimization models, only requiring 80.96 s, greatly improving the running efficiency of the optimization model. This indicates that the model can achieve both high accuracy and good efficiency.

**Figure 11:**
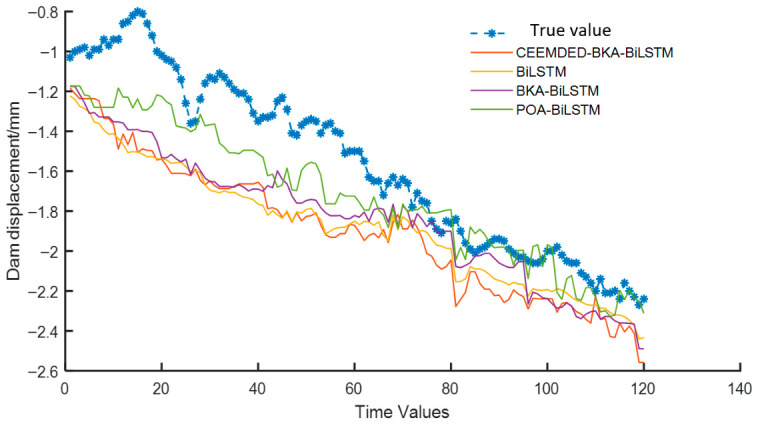
Prediction results of various models at B26-IP-01 measurement point.

To further investigate the reliability and stability of the model, the deformation sequence of measurement point B26-IP-01 was fitted and predicted. The evaluation indicators of the model prediction set are shown in Table 3, and the comparison between the prediction curves of each model and the true values is shown in Figure 11. All results can be found in Supplementary Materials attached to this article.

From Figure 11, the same experimental procedure was applied to monitoring point B26-IP-01 (dam Section 26), and the prediction accuracy evaluation indicators for all models are listed in Table 3. As shown in Figure 11 (prediction curves for B26-IP-01), the CEEMDAN-BKA-BiLSTM model again exhibits the closest agreement with the measured dam deformation values in both magnitude and trend, demonstrating the best prediction performance among all compared models. From the quantitative metrics in Table 3, the MAE, MAPE, MSE, and RMSE of the CEEMDAN-BKA-BiLSTM model are the smallest. Specifically, compared with the other three models (BiLSTM, BKA-BiLSTM, and POA-BiLSTM), the MAE of the proposed model is reduced by 22.4–30.5%, the MAPE by 34.1–40.0%, the MSE by 59.2–67.5%, and the RMSE by 34.1–41.1%. The R^2^ value of the proposed model reaches 0.9427, which is 10.0–14.8% higher than those of the comparison models. These results further confirm that the proposed model not only achieves high prediction accuracy but also maintains stable performance across different dam sections. Regarding computational efficiency, the CEEMDAN-BKA-BiLSTM model requires 92.14 s, which is significantly lower than the BKA-BiLSTM (173.14 s) and POA-BiLSTM (196.21 s) models, although slightly higher than the plain BiLSTM (89.25 s). Considering the substantial improvement in prediction accuracy, the modest increase in computation time is acceptable, and the proposed model still demonstrates a clear advantage in efficiency over other optimization-based models.

### 4.4. Combining SHAP Feature Analysis and Abnormal Cause Inference

**Figure 12:**
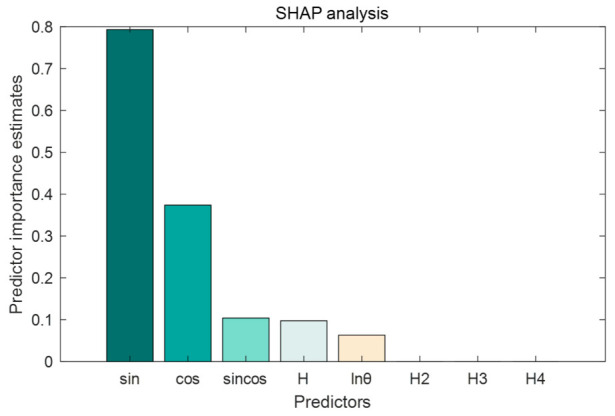
Ranking of the importance of influencing factors.

This article takes the discriminator trained in the model as the explanatory object and obtains the importance ranking of the influencing factors of dam displacement. As shown in Figure 12, the temperature factors sin 2πt/365 and cos 2πt/365 have a high impact on the model prediction, while the impact on other indicators is relatively small.

**Figure 13:**
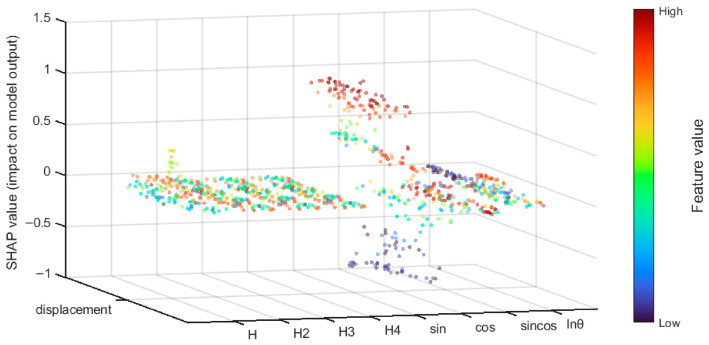
SHAP feature summary.

The SHAP feature summary is shown in Figure 13. When the SHAP value is positive, it indicates that the factor promotes the occurrence of abnormal states; when the SHAP value is negative, it indicates that the factor has an inhibitory effect on the occurrence of abnormal states. The high-value samples (red) of the indicators significantly increase the SHAP value, indicating that fluctuations in these indicators beyond the normal range will directly push the model to determine an abnormal state. The low value samples (purple) are mostly distributed in the SHAP negative area, indicating that when the relevant indicators are in the normal low range, the model tends to determine a normal state, which conforms to the logic of abnormal judgment of stability indicators.

The temperature factor is significantly higher than other features. From the perspective of concrete dam mechanics, the dominant contribution of temperature factors over water pressure factors can be reasonably explained. For concrete dams, periodic ambient temperature variations cause thermal expansion and contraction of the dam concrete and foundation rock, and the temperature field exhibits a significant annual cycle. The case study dam is located in a region with a large annual temperature difference (exceeding 30 °C). Under such conditions, the thermally induced deformation typically accounts for 30–50% of the total annual deformation. In contrast, the water pressure component mainly affects the horizontal displacement of the upstream face, and its influence is relatively localized. The reservoir water level at this hydropower station has a limited annual variation (approximately 15 m). Consequently, the contribution of water pressure factors is smaller than that of temperature factors. This mechanistic interpretation is consistent with classical dam engineering theories and supports the SHAP-based finding that temperature factors dominate the prediction model. The SHAP values of high-value samples (red dense areas) are concentrated in the positive range (>0), suggesting that changes in temperature are the main cause of abnormal dam displacement in the dam environment. In addition, the water level changes and time factors of the dam have a certain impact on the displacement changes of the dam, which cannot be ignored when conducting safety monitoring. The SHAP method enhances the interpretability of the model, and verifies the rationality and reliability of the model anomaly detection process by combining theoretical and practical rules.

## 5. Conclusions

Considering that conventional prediction models are difficult to comprehensively describe the various components of dam deformation, a prediction method integrating interpretable deformation components is proposed. A dam deformation prediction model based on CEEMDED-BKA-BiLSTM is constructed, and the following conclusions are drawn through case analysis:

(1) Multiple regression can be used to separate the temporal components of dam deformation, and the CEEMDAN method can be used to decompose and reconstruct the deformation sequence of the dam, extract the environmental load component and monitoring error component of deformation, while reducing data complexity and improving the interpretability and accuracy of deformation prediction.
(2) The BKA optimization algorithm effectively optimizes the hyperparameters of the BiLSTM model. At measurement point B12-IP-01, the proposed CEEMDAN-BKA-BiLSTM model achieves a test set MAE of 0.2188 mm, RMSE of 0.2627 mm, and R^2^ of 0.9749, which significantly outperforms the standard BiLSTM model (R^2^ = 0.7732). At measurement point B26-IP-01, the proposed model achieves a test set MAE of 0.1430 mm, RMSE of 0.1594 mm, and R^2^ of 0.9427, again substantially better than the comparison models. These results demonstrate that separate prediction and integration of dam deformation components yields significantly higher accuracy, stability, and reliability across different dam sections.
(3) By combining the SHAP interpretation method to quantify the contribution of multiple indicators to abnormal states, we have enhanced the interpretability analysis of the model based on intelligent diagnosis of dam abnormal states, providing a new method for intelligent operation and maintenance of dams that combines high accuracy and transparency.

## Figures and Tables

**Table 1:** Training set fitting accuracy.

Algorithm	MAE/mm	MAPE/%	MSE/mm^2^	RMSE/mm	R^2^
CEEMDED-BKA-BiLSTM	0.1065	0.1643	0.0209	0.1444	0.9956
BiLSTM	0.2031	0.1743	0.0718	0.2679	0.9849
BKA-BiLSTM	0.2041	0.4278	0.0722	0.2688	0.9848
POA-BiLSTM	0.2864	0.3637	0.1392	0.3731	0.9706

**Table 2:** Evaluation indicators for the prediction accuracy of various models at B12-IP-01 measurement point.

Algorithm	*MAE*/mm	*MAPE*/%	*MSE*/mm^2^	*RMSE*/mm	*R* ^2^	Time/t
CEEMDED-BKA-BiLSTM	0.2188	0.5121	0.0690	0.2627	0.9749	80.96
BiLSTM	0.6503	0.6748	0.6243	0.7901	0.7732	68.04
BKA-BiLSTM	0.4460	0.8223	0.2821	0.5311	0.8975	169.54
POA-BiLSTM	0.4933	0.7269	0.3612	0.6010	0.8688	189.23

**Table 3:** Evaluation indicators for the prediction accuracy of various models at B26-IP-01 measurement point.

Algorithm	*MAE*/mm	*MAPE*/%	*MSE*/mm^2^	*RMSE*/mm	*R* ^2^	Time/t
CEEMDED-BKA-BiLSTM	0.1430	0.0836	0.0238	0.1594	0.9427	92.14
BiLSTM	0.1923	0.1320	0.0594	0.2438	0.8546	89.25
BKA-BiLSTM	0.1842	0.1268	0.0584	0.2418	0.8569	173.14
POA-BiLSTM	0.2057	0.1394	0.07326	0.2706	0.8208	196.21

## Data Availability

All relevant data are within the manuscript and its Supplementary Materials.

## Supplementary Materials

The following supporting information can be downloaded at: https://www.mdpi.com/article/10.3390/s26082495/s1.

## Abbreviations

The following abbreviations are used in this manuscript:

BKA	Black-Winged Kite Optimization Algorithm
CEEMDAN	Complementary ensemble empirical mode decomposition with adaptive noise
BiLSTM	Bidirectional Long Short Term Memory
SHAP	SHapley Additive exPlanations
